# Estimating the impact of child and early adolescent depression on subsequent educational attainment: secondary analysis of an existing data linkage

**DOI:** 10.1017/S2045796021000603

**Published:** 2021-11-25

**Authors:** A. Wickersham, T. Ford, R. Stewart, J. Downs

**Affiliations:** 1 Institute of Psychiatry, Psychology & Neuroscience, King's College London, London, UK; 2 Department of Psychiatry, University of Cambridge, Cambridge, UK; 3 South London and Maudsley NHS Foundation Trust, London, UK

**Keywords:** Adolescence, child psychiatry, depression, epidemiology, performance

## Abstract

**Aims:**

Depression is thought to be associated with lower subsequent educational attainment during school. But, without longitudinal studies which take account of prior attainment and other potential confounders, estimates of the impact of clinically recognised depression in childhood and early adolescence are unknown. We investigated whether a clinical diagnosis of depression is associated with lower subsequent educational attainment, and whether the association is modified by gender, ethnicity and socioeconomic status.

**Methods:**

We conducted a secondary analysis of an existing administrative data linkage between national educational data and a large mental healthcare provider in London, UK (2007–2013). Depression diagnosis before age 15 (exposure) was measured from electronic health records, and subsequent educational attainment at age 15–16 (outcome) was measured from educational records. We fitted logistic regression models and adjusted for gender, ethnicity, socioeconomic status, relative age in school year, neurodevelopmental disorder diagnosis and prior attainment. We investigated effect modifiers using interaction terms.

**Results:**

In total, *n* = 63 623 were included in analysis, of whom *n* = 242 had record of a depression diagnosis before age 15. Depression was associated with lower odds of subsequently achieving expected attainment levels in national exams, after adjustment for all covariates (odds ratio = 0.60, 95% confidence interval = 0.43 to 0.84, *p* = 0.003). There was no evidence that gender, ethnicity or socioeconomic status modified this association.

**Conclusions:**

These findings support a relationship between depression and lower subsequent educational attainment. This highlights the need for tailored educational interventions to support children and adolescents with depression, particularly in the lead up to key educational milestones.

## Introduction

Incidence of child and adolescent depression rapidly increases during mid-teenage years (Thapar *et al*., 2012; Ghandour *et al*., 2019). In many countries, high-stake school exams often take place during this developmental period that influence future educational and occupational trajectories. With symptoms of depression including low mood, reduced energy, loss of interest in activities and lower concentration, this disorder can have a negative impact on such school outcomes (Thapar *et al*., 2012).

A large, population-based cohort study recently showed a strong, negative association between receiving a unipolar depression diagnosis before age 16 and subsequent exam grades among both male and female pupils (Dalsgaard *et al*., 2020). However, they did not adjust for some key variables thought to be associated with both emotional disorders and school performance, such as ethnicity and socioeconomic status (Strand, 2014; Sadler *et al*., 2018). Moreover, they (and many other longitudinal studies of this relationship) were unable to control for earlier attainment in these analyses, which limits conclusions on the direction of effect; low school performance at a previous timepoint could potentially underlie both the onset of depression and later attainment. Indeed, a recent study suggested that depression was associated with a general decline in attainment over time (Rahman *et al*., 2018), although the timing of depression diagnosis in relation to such declines remain unclear (Wickersham *et al*., 2020*a*).

The apparent scarcity of studies which adjust for earlier levels of school performance led one narrative review to suggest that there is no indication of any causal relationship between internalising problems (such as depression) and academic performance (Suldo *et al*., 2014). A recent systematic review and meta-analysis was able identify a few studies which demonstrated a weak, negative association between depression symptoms, as identified from brief screening instruments, and subsequent attainment even after making such adjustments (Wickersham *et al*., 2021). However, there were no evaluations of clinically diagnosed depression and attainment during adolescence which adjusted for prior school performance. This was reflective of an earlier review, which similarly highlighted a need for community studies on this association using clinical diagnoses (Riglin *et al*., 2014). This limits the known implications for clinical groups who are recognised by mental health services as having a depression disorder, among whom psychopathology severity is known to be much greater, but who are also more likely to be in receipt of evidence-based support (Ford *et al*., 2008).

Given the unclear findings on this association, a similar lack of clarity about effect modifiers is perhaps unsurprising. Studies have shown mixed findings on whether sociodemographic characteristics moderate the association between depression and attainment (Wickersham *et al*., 2021), despite plausible mechanisms. To address these gaps in the literature, the research questions investigated in the current study were:

1. Is a clinical diagnosis of depression associated with lower subsequent educational attainment?
2. Is this association modified by gender, ethnicity and socioeconomic status?

We hypothesised that, compared to the general school-aged population, children and adolescents who received a diagnosis of depression would have lower odds of subsequently performing at expected levels in national exams. We adjusted for prior school performance in order to address the directional aspect of this hypothesis. Previous findings are mixed on the role of sociodemographic characteristics as modifiers for this association; therefore, we did not formulate any directional hypotheses for our second research question.

## Method

### Study design and data sources

The analysis reported here was an historical, retrospective cohort study pre-registered on the Open Science Framework in June 2020 (Wickersham *et al*., 2020*b*). Reporting follows RECORD guidelines (online Supplementary Table S1). This was a secondary analysis of an existing individual-level data linkage between the National Pupil Database (NPD) and the Clinical Record Interactive Search (CRIS) at South London and Maudsley NHS Foundation Trust (SLaM).

The NPD is a national data resource containing educational records for pupils in England's state schools, including attainment data for Year 6 Standard Assessment Tests (SATs, typically undertaken at ages 10–11) and General Certificates of Secondary Education (GCSEs, typically undertaken at ages 15–16) (Jay *et al*., 2019). CRIS provides research access to pseudonymised electronic health records for SLaM's secondary mental health services, including Child and Adolescent Mental Health Services (CAMHS) (Perera *et al*., 2016).

The CRIS–NPD linkage process has been described in detail elsewhere (Downs *et al*., 2017, 2019). In brief, the linkage was conducted for referrals to SLaM between 2007 and 2013, with children and adolescents aged between 4 and 18 years. A fuzzy deterministic matching procedure was developed based on personal identifiers in CRIS, which were sent to the Department for Education for linkage to the NPD. The match rate was 82.5%, and while individual characteristics such as age, ethnicity and deprivation were associated with successful linkage, adjusting for these linkage biases did not appear to materially change the strength or direction of results arising from analyses using this linkage.

As a result of this data linkage, we had access to educational records for *n* = 276 655 pupils, including local pupils who were not referred to SLaM, and pupils who were referred to SLaM from both inside and outside the catchment area.

### Study setting

SLaM is the provider for secondary mental healthcare services for a catchment of four south London boroughs: Croydon, Lambeth, Lewisham and Southwark. The catchment area comprises a population of approximately 1.3 million individuals. Compared to the rest of London and England, the catchment area has a higher proportion of minority ethnic groups, and a higher proportion of deprived areas (Office for National Statistics, 2013, 2019, 2020).

SLaM CAMHS comprises community outpatient CAMHS accessible to residents in each of the four boroughs in SLaM's catchment area, national and specialist outpatient services which are also accessible to patients from outside the catchment area, and inpatient services. Within each borough's community outpatient service there is a range of multi-disciplinary teams meeting different levels and types of need. They variously offer assessment and treatment for mild, moderate, severe and complex mental health disorders in children and adolescents within the 0–18 year age range.

### Study sample

Pupils were eligible for inclusion in the current analysis if they were residents of the local catchment area, as this was the geographical area for which we had a comparison group of pupils not referred to SLaM for a depression diagnosis. Their educational records needed to contain available GCSE attainment data, as this was the outcome variable of interest, and they needed to be aged 15 years or older at the start of the academic year in which GCSE results were recorded, in order to ensure that the depression diagnosis exposure occurred before the attainment outcome. As is standard practice for these educational data (Duckworth and Schoon, 2010), and to avoid missing not at random data patterns, we also excluded pupils who were missing attainment data from either their Maths or English Year 6 SATs due to absence or disapplication. Disapplied pupils are those who are unable to participate in national exams, even with additional arrangements, usually because of special educational needs which make the national curriculum unsuited to them.

### Outcome: GCSE attainment

In the UK, GCSEs are high-stake exams which are often considered by institutions when applying for further education and university. GCSE attainment was extracted from the NPD as a binary variable indicating whether pupils were awarded five or more GCSEs and equivalents at grades A* to C, including GCSE English and Maths (Department for Education). At the time of our observation window, this indicator of attainment was seen as the expected minimum attainment threshold that pupils should reach, and was used to assess school effectiveness. Accordingly, it was reported in the Department for Education's annual statistical releases on school performance in England (Department for Education, 2011*b*).

### Exposure: depression diagnosis

Depression diagnosis was extracted from CRIS as a binary variable, indicating whether a first depression diagnosis was recorded by SLaM secondary mental health services before age 15 in structured primary or secondary diagnosis fields. Depression diagnosis was defined as any International Statistical Classification of Diseases and Related Health Problems – 10th Revision (ICD-10) F32*x* or F33*x* depressive disorder diagnosis.

### Covariates

Gender, ethnicity and birth month were coded from multiple NPD and CRIS datasets, assigning from a subsequent dataset if missing from the previous, in the following order: school census, key stage 1, key stage 2, key stage 4, absence data tables and CRIS. Gender was coded as a binary variable (male or female), and ethnicity as a categorical variable (White, Black or Other). Birth month was transformed into a categorical variable indicating relative age within the school year, including autumn-born (September to December), spring-born (January to April) and summer-born (May to August) (Goodman *et al*., 2003). Free School Meal (FSM) eligibility was coded as a binary variable indicating whether pupils had ever been recorded as being eligible for FSM (eligible or ineligible) in school census or absence data tables. FSM eligibility is determined based on parental income, and is therefore commonly used as a proxy indicator of socioeconomic disadvantage in the UK (Gorard, 2012).

Neurodevelopmental disorder was extracted from CRIS, and was defined as any ICD-10 diagnosis of intellectual disability (F70*x*–F79*x*), pervasive developmental disorder (F84*x*) or hyperkinetic disorder (F90*x*), as recorded at any age in structured primary or secondary diagnosis fields. Attainment in Year 6 SATs was extracted from the NPD as a binary variable, indicating whether pupils achieved the expected attainment threshold of Level 4 or above in both English and Maths assessments.

We generated a birth year variable for use in missing data investigations. For each pupil, we took the mean average birth year across estimates derived from CRIS, school census and age at each key stage. Finally, we combined the generated birth month and birth year variables to estimate age at first depression diagnosis (birth date was taken as the first of the month).

### Statistical analysis

Prior to the main analysis, missing data were identified in some covariates, and likelihood of having a complete record was associated with the remaining fully observed variables and with birth year (used as a proxy for changes in which variables were recorded in the NPD over different years). These investigations generally supported a missing at random assumption. Therefore, inverse probability weights were generated using fully observed variables and birth year, and all analyses were limited to complete records, but weighted according to each pupil's likelihood of having a complete record (Seaman and White, 2013).

To investigate our first research question, we initially conducted univariable logistic regression to estimate the unadjusted association between depression (exposure) and GCSE attainment (outcome). We then adjusted for potential confounders in a series of multivariable logistic regressions, first adjusting for gender, ethnicity, FSM eligibility, relative age in the school year and neurodevelopmental disorder, then additionally adjusting for Year 6 SATs attainment. Finally, to investigate our second research question, we re-ran the fully adjusted multivariable logistic regression model three times, each time adding an interaction term between depression and gender, ethnicity or FSM eligibility, and tested these interaction terms using Wald tests. Inferences about the strength of evidence for the associations under study were made based on the magnitude of effect estimates, confidence intervals (CIs) and two-tailed *p* values (*p* < 0.05). All analyses were conducted in R 3.5.1 (R Core Team, 2021).

## Results

In total, *n* = 83 231 pupils were eligible for inclusion in analysis. Of these, 21.7% (*n* = 18 072) were missing ethnicity data, 2.0% (*n* = 1642) were missing FSM eligibility data and 10.4% (*n* = 8678) were missing Year 6 SATs data (missingness stratified by GCSE attainment can be found in online Supplementary Table S2). The total number of pupils with complete records for all the variables of interest, and therefore included in the final analytical sample, was *n* = 63 623. Study sample flow diagrams can be found in online Supplementary Figs S1 and S2, and the characteristics of pupils who were eligible *v.* those included in the final analytical sample are compared in online Supplementary Table S3.

The majority of the final analytical sample was White and had never been eligible for FSM, while gender and relative age in the school year were evenly balanced (Table 1). Most achieved five or more A* to C GCSE and equivalent grades including English and Maths, and most had been awarded a Level 4 and above in both English and Maths Year 6 SATs. A small proportion had received diagnoses of depression before age 15, or of a neurodevelopmental disorder at any age.

**Table 1.** Study variables by GCSE attainment (unweighted frequencies and percentages)

	<5 A* to C grades (*n* = 27 823)	⩾5 A* to C grades (*n* = 35 800)	Total (*n* = 63 623)
Depression diagnosis before age 15			
No	27 683 (99.5%)	35 698 (99.7%)	63 381 (99.6%)
Yes	140 (0.5%)	102 (0.3%)	242 (0.4%)
Gender			
Female	12 695 (45.6%)	19 402 (54.2%)	32 097 (50.4%)
Male	15 128 (54.4%)	16 398 (45.8%)	31 526 (49.6%)
Ethnicity			
White	12 175 (43.8%)	15 048 (42.0%)	27 223 (42.8%)
Black	10 459 (37.6%)	12 102 (33.8%)	22 561 (35.5%)
Other	5189 (18.7%)	8650 (24.2%)	13 839 (21.8%)
Ever eligible for FSM			
No	13 818 (49.7%)	25 044 (70.0%)	38 862 (61.1%)
Yes	14 005 (50.3%)	10 756 (30.0%)	24 761 (38.9%)
Relative age in school year			
Autumn-born	8841 (31.8%)	12 614 (35.2%)	21 455 (33.7%)
Spring-born	8875 (31.9%)	11 428 (31.9%)	20 303 (31.9%)
Summer-born	10 107 (36.3%)	11 758 (32.8%)	21 865 (34.4%)
Neurodevelopmental diagnosis at any age			
No	26 697 (96.0%)	35 594 (99.4%)	62 291 (97.9%)
Yes	1126 (4.0%)	206 (0.6%)	1332 (2.1%)
Year 6 SATs attainment: Level 4 in both English and Maths			
No	16 768 (60.3%)	3387 (9.5%)	20 155 (31.7%)
Yes	11 055 (39.7%)	32 413 (90.5%)	43 468 (68.3%)

The median age of first depression diagnosis was 14 years (range = 9–14 years, interquartile range = 13–14 years). Compared to those without a depression diagnosis, those who received a depression diagnosis before age 15 were more frequently female, White, eligible for FSM, summer-born and diagnosed with a neurodevelopmental disorder (online Supplementary Table S4). The majority also achieved a Level 4 and above in both English and Maths Year 6 SATs (65.3%, *n* = 158), similar to those without a depression diagnosis (68.3%, *n* = 43 310). However, only 42.1% (*n* = 102) of those with a depression diagnosis before age 15 achieved five or more A* to C GCSE and equivalent grades including English and Maths, compared to 56.3% (*n* = 35 698) of those without depression.

Results of univariable logistic regression suggested that children and adolescents who received a diagnosis of depression before age 15 were at 40% lower odds of achieving five GCSE or equivalent A* to C grades including English and Maths, as compared to those who did not receive a depression diagnosis (odds ratio = 0.60, 95% CI = 0.47 to 0.78, *p* < 0.001). This association persisted after adjusting for covariates, including prior attainment in Year 6 SATs (Table 2). Finally, Wald tests for interaction terms added to the fully adjusted multivariable logistic regression model did not show any evidence for interactions between depression and gender (*χ*^2^(1) = 0.89, *p* = 0.345), ethnicity (*χ*^2^(2) = 2.54, *p* = 0.281) or FSM eligibility (*χ*^2^(1) = 0.91, *p* = 0.340) in predicting GCSE attainment.

**Table 2.** Results of logistic regression models predicting GCSE attainment (<5 A* to C grades was the reference category) (*n* =  63 623)

	Model 1^a^	*p*	Model 2^b^	*p*	Model 3^c^	*p*
	OR (95% CI)		OR (95% CI)		OR (95% CI)	
Depression diagnosis before age 15						
No	Reference	–	Reference	–	Reference	–
Yes	0.60 (0.47 to 0.78)	<0.001	0.69 (0.52 to 0.92)	0.011	0.60 (0.43 to 0.84)	0.003
Gender						
Female	–	–	Reference	–	Reference	–
Male	–	–	0.72 (0.69 to 0.74)	<0.001	0.72 (0.70 to 0.75)	<0.001
Ethnicity						
White	–	–	Reference	–	Reference	–
Black	–	–	1.02 (0.99 to 1.06)	0.236	1.26 (1.20 to 1.31)	<0.001
Other	–	–	1.48 (1.42 to 1.55)	<0.001	1.57 (1.49 to 1.65)	<0.001
Ever eligible for FSM						
No	–	–	Reference	–	Reference	–
Yes	–	–	0.42 (0.41 to 0.44)	<0.001	0.53 (0.51 to 0.55)	<0.001
Relative age in school year						
Autumn-born	–	–	Reference	–	Reference	–
Spring-born	–	–	0.91 (0.87 to 0.95)	<0.001	1.01 (0.97 to 1.06)	0.604
Summer-born	–	–	0.82 (0.78 to 0.85)	<0.001	1.01 (0.97 to 1.06)	0.621
Neurodevelopmental diagnosis at any age						
No	–	–	Reference	–	Reference	–
Yes	–	–	0.17 (0.15 to 0.20)	<0.001	0.28 (0.24 to 0.33)	<0.001
Year 6 SATs attainment: Level 4 in both English and Maths						
No	–	–	–	–	Reference	–
Yes	–	–	–	–	13.52 (12.94 to 14.13)	<0.001

OR, odds ratio; CI, confidence interval.

a Model 1: Unadjusted association between depression and GCSE attainment.

b Model 2: Adjusted for gender, ethnicity, FSM eligibility, relative age in school year and neurodevelopmental diagnosis.

c Model 3: Further adjusted for Year 6 SATs attainment.

## Discussion

The findings from this study supported our first hypothesis that receiving a depression diagnosis was associated with lower subsequent educational attainment. However, we did not find that the association was modified by gender, ethnicity or socioeconomic status.

Our findings are consistent with previous studies showing an association between depression diagnosis and subsequent attainment during school (Dalsgaard *et al*., 2020; Wickersham *et al*., 2021). Moreover, this association persisted after adjusting for potentially confounding sociodemographic characteristics, and strengthened after further adjusting for prior attainment, perhaps because prior attainment was similar between those with and without depression after adjusting for all other variables. Our findings support a previous longitudinal trajectory analysis conducted in a subset of this cohort, which found that depression diagnosis either seemed to coincide with or precede a significant drop in educational attainment (Wickersham *et al*., 2020*a*). The findings are also similar to a study conducted in Sweden showing that those with a depression diagnosis were less likely to graduate from higher education during adulthood, even after adjusting for prior attainment, although they only found this among males (Jonsson *et al*., 2010).

Overall, our findings suggest that depression is a significant but highly modifiable risk factor for lower educational attainment, with those who received a depression diagnosis at 40% lower odds of achieving the minimum attainment thresholds which are expected at the end of secondary education. Gender, ethnicity and socioeconomic status did not modify the association, suggesting that the association is pervasive across sociodemographic backgrounds. In the UK, results achieved in these exams are often considered by institutions when applying for further education and university, and so can open or close opportunities for future study. With low attainment in turn associated with later risk for health problems, homelessness and unemployment, these associations could set in motion a vicious circle of poor health and loss of occupation opportunity (Caspi *et al*., 1998; Almquist, 2013; Brakenhoff *et al*., 2015).

It has long been recognised that fostering positive child and adolescent mental health may optimise school outcomes, including higher academic attainment and better attendance (Bonell *et al*., 2014). Our findings support this conclusion, suggesting that not only should children and adolescents with depression receive additional educational support if required, but also that effective interventions for depression might improve their later educational outcomes. CAMHS clinicians should work with educational practitioners to minimise the impact of depression on attainment at school and work in partnership with them to address other factors which might be important mechanisms in the association, such as concentration, motivation and retention. To empirically establish these mechanisms, and to establish whether CAMHS depression treatments can improve school outcomes, it would be important for future trials of depression interventions among children and adolescents to include such educational outcome measures. To date, these outcomes are not commonly included in trials of mental health interventions, but have shown interesting results when such outcomes are included (Wyman *et al*., 2010; Ougrin *et al*., 2018). Other plausible mechanisms in the school environment should also be further explored, such as peer victimisation (Liu *et al*., 2018) and school absence (Hughes *et al*., 2021). Children and adolescents with depression may also benefit from other support networks outside of school, from deferring or staggering their exams until they have undergone treatment for their depression, or from pursuing alternative educational pathways which place less emphasis on exam performance (Health Foundation, 2019).

This study benefits from a large sample of pupils with routinely collected clinical and educational records. This overcomes known issues with self- or informant-reported attainment (Kuncel *et al*., 2005), and biases associated with recruitment and data collection in more traditional longitudinal cohort studies. However, some limitations remain. Biases such as misclassification bias remain possible, and we were unable to conduct validation work as part of this study. We also excluded pupils who were absent or disapplied from either Year 6 SATs assessment, because such assessments are scored as missing by the Department for Education and thus their inclusion can introduce missing not at random data patterns. However, this in turn introduces some selection bias, as those who are disapplied or absent from Year 6 SATs assessments are less likely to meet expected GCSE attainment thresholds.

Focusing on children and adolescents who receive a clinical diagnosis of depression from CAMHS also precludes generalisation to those who do not access mental health services, or who have milder sub-clinical depression symptoms – indeed, a study of depression symptoms in another London-based community sample could not identify an association with GCSE attainment after adjusting for prior school performance (Rothon *et al*., 2009). In the community, there are a large number of children and adolescents who would meet diagnostic criteria for depression but are either accessing help from other sources (such as primary care or schools) (Potter *et al*., 2012), or are not in contact with services (Neufeld *et al*., 2017). Compared to other groups with depression in the community, our CAMHS sample may have had more severe and complex mental health problems, although they are also likely to have been in receipt of some evidence-based treatment (NICE, 2019). These groups could also have different comorbidity rates. In our CAMHS sample, 9.9% of children and adolescents with a depression diagnosis also received a neurodevelopmental disorder at any age; this is reasonably comparable to a national study where, among 5–19 year olds meeting criteria for emotional disorders, 11.0% also had speech or language problems, and 8.4% also had coordination difficulties (Sadler *et al*., 2018). Nonetheless, understanding the role of identification, comorbidities, treatment type, treatment length and treatment response in the association between depression and school performance would be interesting areas for future research, as would the role of special educational needs provision.

In order to support a direction of effect, we ensured that depression diagnosis was recorded before age 15, and that only pupils who were aged 15 or older at the start of their GCSE academic year were included. Despite this, it remains possible that some assessments were taken early, although it should be noted that such early entries usually take in place in school years consistent with pupils being 15 years or older, such that depression diagnosis should still precede exam taking in most cases (Department for Education, 2011*a*). Although widely used as a proxy indicator for deprivation, FSM eligibility is also an imperfect measure; many children are entitled to FSM but do not have an FSM claim made on their behalf, such that they are not recorded as eligible for FSM (Iniesta-Martinez and Evans, 2012). Other deprivation measures, such as the Income Deprivation Affecting Children Index, could also be considered in future research. We also acknowledge that due to small cell sizes, we grouped the ethnicity covariate into White, Black or Other – conducting more granular investigations of ethnicity would be an important area for future research. Similarly, the low number of pupils with a depression diagnosis resulted in some small cell sizes for analyses of effect modifiers; future research could seek to replicate these findings in a cohort with a higher proportion of depression diagnoses.

In a large cohort of children and adolescents with linked health and education data, our findings showed a negative association between depression diagnosis before age 15 and subsequent educational attainment. This association persisted after adjusting for key sociodemographic characteristics and prior educational attainment. Gender, ethnicity and socioeconomic status did not modify the association, suggesting that the relationship is pervasive for children from many different sociodemographic backgrounds. These findings highlight the critical need for educational and mental health support among children and adolescents with depression, particularly in the lead up to key educational milestones.

## Data Availability

The data cannot be made publicly available, but can be accessed with permissions from both the Department for Education and South London and Maudsley NHS Foundation Trust. At the time of submission, JD and AW have full and ongoing access to the data.

## Supplementary material

For supplementary material accompanying this paper visit http://dx.doi.org/10.1017/S2045796021000603. click here to view supplementary material
